# Constipation Is Linked to Neuroinflammation in Early Parkinson's Disease

**DOI:** 10.1002/mds.70102

**Published:** 2025-11-13

**Authors:** Marta Camacho, Julia C. Greenland, Alexander R.D. Peattie, Lennart R.B. Spindler, Jonathan Holbrook, Lakmini Kahanawita, Tim D. Fryer, Young T. Hong, Caroline H. Williams‐Gray

**Affiliations:** ^1^ Department of Clinical Neurosciences University of Cambridge Cambridge UK; ^2^ Dementia Discovery Fund London UK; ^3^ Department of Clinical Neurosciences Wolfson Brain Imaging Centre, University of Cambridge Cambridge UK

**Keywords:** Parkinson's disease, PET imaging, inflammation, constipation

## Abstract

**Background:**

Constipation is a risk factor for the onset and accelerated progression of Parkinson's disease (PD), but the mechanisms underlying this association are unknown. Neuroinflammation in PD has been demonstrated in postmortem and neuroimaging studies; however, its relationship with constipation has not been investigated.

**Methods:**

We used ^11^C‐PK11195 positron emission tomography (PET) to index neuroinflammation in a cohort of 27 people with early‐stage PD. The Gastrointestinal Dysfunction Scale for PD (GIDS‐PD) was used to assess gut symptom severity. Matched blood and cerebrospinal fluid (CSF) samples were collected.

**Results:**

Higher GIDS‐PD constipation scores were associated with ^11^C‐PK11195 binding in whole‐brain gray matter, frontal, parietal, temporal, and occipital lobes, and multiple subcortical and cortical regions. GIDS‐PD Constipation scores were also significantly associated with higher CSF lymphocyte count and blood T helper 1 (Th1) cell and Th17‐like Th1 cells.

**Conclusions:**

Constipation in early PD is associated with widespread neuroinflammation, suggesting a possible mechanism underlying the association between constipation and faster PD progression. © 2025 The Author(s). *Movement Disorders* published by Wiley Periodicals LLC on behalf of International Parkinson and Movement Disorder Society.

People with constipation are at greater risk of developing Parkinson's disease (PD),^1^ and early constipation in PD patients has been associated with accelerated disease progression, particularly the development of PD dementia (PDD).^2^, ^3^, ^4^, ^5^ Despite the recent growth in research efforts to understand the role of the gut–brain axis in the pathophysiology of PD, the mechanisms underlying the relationship between constipation and brain pathology are still unknown. There are several mechanisms through which gut dysfunction might impact PD pathology, including the neuroendocrine system, the vagus nerve, microbiota‐derived metabolites, and the immune system.^6^ Colonic alpha‐synuclein is associated with chronic constipation^7^, ^8^ and patients with constipation have been found to have increased intestinal permeability and an activated peripheral immune response.^9^ Likewise, local intestinal inflammation may alter the contractile activity of intestinal smooth muscle,^10^ inducing delayed motility, which, in turn, can aggravate mucosal inflammation, increase intestinal permeability, and contribute to peripheral inflammation.^7^, ^11^ Immune alterations are well described in the blood in PD, with altered cytokine levels,^12^, ^13^ monocyte^14^, ^15^ and lymphocyte phenotype^16^, ^17^, ^18^ which may influence the central nervous system (CNS) via various crosstalk mechanisms between the CNS and periphery.^19^ Both rodent and pediatric enteric alpha‐synuclein depositions have been linked to immune cell activation in the CNS.^20^ The integrity of the blood–brain barrier may be disrupted in PD,^21^ and there is evidence of impaired meningeal lymphatic drainage^22^ and increased lymphocyte trafficking into the CNS.^23^, ^24^ Moreover, microglial activation has been demonstrated in the PD brain in postmortem studies,^23^, ^24^, ^25^, ^26^ consistent with the neuroinflammation found in vivo using positron emission tomography (PET) with radioligands for the translocator protein (TSPO), expressed in microglia, such as ^11^C‐PK11195.^27^, ^28^, ^29^ Both ^11^C‐PK11195 PET and neuropathological studies have shown that neuroinflammation is further increased in PDD cases,^24^, ^27^ with increased microglial activation and infiltration of T lymphocytes in cognition‐relevant regions at postmortem.^24^ Neuroinflammation has also been reported in other dementias,^30^, ^31^ mild cognitive impairment,^32^ and in people with rapid eye movement (REM) sleep behavior disorder,^33^, ^34^ who are at high risk of developing PD and are likely to experience constipation.^35^ Moreover, several longitudinal studies have shown that constipation in early PD is associated with faster cognitive decline and PDD onset.^2^, ^3^, ^4^, ^5^, ^36^, ^37^ Therefore, it is possible that constipation‐related gut alpha‐synuclein deposition, as well as microbiome and associated metabolite changes, may contribute to low‐grade gut and systemic inflammation in PD, in turn exacerbating brain inflammation and neurodegeneration, leading to more rapid disease progression.^7^ This relationship is likely bidirectional, with local gastrointestinal (GI) inflammation also contributing to impaired GI motility and aggravation of constipation.

However, to date, studies have not investigated the relationship between constipation and CNS inflammation in individuals with PD. We hypothesized that (i) PD patients with constipation would have higher levels of brain inflammation compared to those without constipation; (ii) the degree of neuroinflammation would correlate with constipation scores; and (iii) regional neuroinflammation would be associated with early deficits in cognitive function. We also explored correlations between constipation and immune cells in the cerebrospinal fluid (CSF) to assess its association with other measures of CNS immune activation, as well as in the peripheral immune system.

## Methods

Ethical approval was obtained from the London‐Westminster Research Ethics Committee (reference: 19/LO/1705) and Yorkshire & the Humber—Bradford Leeds Research Ethics Committee (19/YH/0198), and the study was approved by the UK Administration of Radioactive Substances Advisory Committee (ARSAC).

### Participants

This study utilized clinical data, ^11^C‐PK11195 brain PET, and biosamples collected at baseline from a subset of participants enrolled in a clinical trial of immunosuppressant therapy for early‐stage PD: “Azathioprine immunosuppression and disease modification in Parkinson's disease (AZA‐PD): a randomized double‐blind placebo‐controlled phase II trial.”^38^ Participants were recently diagnosed PD cases (≤3 years disease duration), aged between 50 and 80 years, and fulfilling UK PD Brain Bank Criteria for idiopathic PD. Exclusion criteria included the presence of chronic inflammatory or autoimmune disorders, current or latent infection, a solid organ malignancy within the previous 5 years, any previous hematological malignancy, vaccinations in the preceding month, use of anti‐inflammatory/immune‐modulating medications, a diagnosis of dementia according to the Movement Disorder Society (MDS) PDD criteria, and significant psychiatric disturbance. Exclusion criteria for CSF collection were the use of an anticoagulant or clopidogrel, papilledema on fundoscopy or a focal neurological deficit on examination. Data used in this study were collected over two visits, which occurred prior to initiation of trial treatment: a clinical/neuropsychological assessment and a ^11^C‐PK11195 PET‐MR scan, with a maximum interval of 6 weeks between them.

### Clinical and Neuropsychological Assessment

Participants were clinically assessed for comorbid conditions and medication history. PD cases underwent standardized assessments of motor and cognitive function, including the MDS–Sponsored Unified Parkinson's Disease Rating Scale (MDS‐UPDRS, completed in the ‘OFF’ medication state), Addenbrooke's Cognitive Examination III (ACE‐III), the Geriatric Depression Scale (GDS‐15) for depression symptom severity, and the Gastrointestinal Dysfunction Scale for Parkinson's disease (GIDS‐PD), with a GIDS‐PD constipation score ≥ 9 used to stratify participants as “constipated” versus “nonconstipated.”^39^ Levodopa‐equivalent daily dose (LEDD) was calculated using an adaptation of the Tomlinson et al formula.^38^ The number of comorbidities at baseline was quantified in terms of the number of organ systems affected using the Cumulative Illness Rating Scale (CIRS).

### PET Imaging and Analysis

PET scans were performed using a GE SIGNA PET/MR scanner (GE Healthcare). List‐mode PET data were acquired for 75 minutes following injection of ^11^C‐PK11195 (405 ± 78 MBq). The PET data were histogrammed into 55 time frames and reconstructed into images (128 × 128 × 89 matrix; 2.0 × 2.0 × 2.8 mm voxel size) using a time‐of‐flight version of ordered subsets expectation maximization^40^ with 16 subsets, 6 iterations, and no smoothing. Attenuation correction used a pseudo‐computed tomography (CT) generated by a multisubject atlas method^41^ from T1‐weighted BRAVO magnetic resonance imaging (MRI) acquired during PET data acquisition (192 × 512 × 512 matrix interpolated to a 192 × 280 × 280 matrix with 1.0 mm isotropic voxel size), together with an improved MRI head coil attenuation template.^42^ Image reconstruction also included corrections for random coincidences, dead time, normalization, scattered coincidences, radioactive decay, and sensitivity.

SPM12 (https://www.fil.ion.ucl.ac.uk/spm/software/spm12/) was used to realign each dynamic PET image series and coregister each realigned dynamic PET image series to the BRAVO MR image from the same scan. To estimate specific tracer binding, ^11^C‐PK11195 PET data were analyzed with the simplified reference tissue model (SRTM)^43^ to quantify binding potential relative to a nondisplaceable compartment (BP_ND_). The reference region was estimated with supervised cluster analysis, with correction for vascular binding included in the model.

For regional analysis, an adapted version of the n30r83 Hammersmith atlas (http://brain-development.org) was transformed to each BRAVO MR using ANTs (https://picsl.upenn.edu). This atlas includes 43 bilateral regions of interest (ROIs) that are established as important in PD histopathological studies. Additional lobar ROIs (frontal, temporal, parietal, occipital, cingulate, and cerebellum) were defined by aggregation of the atlas ROIs, and a whole‐brain gray matter ROI was defined by applying a 50% lower threshold to the SPM12 gray matter probability map smoothed to PET spatial resolution. The time‐activity curve of each ROI was corrected for CSF contamination through division with the mean sum of gray and white matter probabilities in the ROI, with both probability maps smoothed to PET spatial resolution. SRTM was then applied to the CSF‐corrected ROI time‐activity curves.

### Measurement of Immune Cells and Inflammatory Markers in Blood and CSF

Venous blood samples were collected in S‐Monovette tubes, including a lithium heparin sample for peripheral blood mononuclear cell (PBMC) extraction, a serum sample for analysis of inflammatory cytokines and C‐reactive protein (CRP), and an ethylenediaminetetraacetic acid (EDTA) sample for full blood count (FBC) analysis. CRP and FBC were measured by Addenbrooke's hospital pathology labs. Serum samples were allowed to clot for 15 minutes prior to centrifugation at 2000 rpm for 15 minutes. Serum was removed and stored in 200 μL aliquots at −80°C until inflammatory marker assays were performed.

A subset of eligible participants consented to a lumbar puncture for CSF collection (an optional part of the AZA‐PD trial protocol). Between 5 and 10 mL of CSF was collected, immediately placed on ice, spun at 300 g for 10 minutes at 4°C for separation of immune cells for flow cytometric analysis, and the resulting supernatant was stored in 200 μL aliquots at −80°C until cytokine assays were performed. PBMCs were extracted by centrifugation over a Ficoll gradient, washed with phosphate‐buffered saline, and blocked using 2% mouse serum. Staining with fluorochrome‐conjugated monoclonal antibodies for flow cytometry was performed on the day of collection to identify lymphocyte (CD3+, CD8+, CD4+) populations, including T helper cell subsets (Th1, Th2, Th17, Th1/17, and Treg), and monocyte (classical, intermediate, and nonclassical) populations. For flow cytometry panels and detailed methods, including the flow cytometry gating strategy, please refer to Greenland et al.^19^ The number of cells per mL of CSF was calculated by applying the total number of live CD45+ gated events from the entire sample and dividing by the volume of CSF collected.

Participants with CRP > 10 μg/mL were excluded from the analysis due to the presumed presence of a concurrent infection or inflammatory disorder. A panel of inflammation‐related markers was measured in the serum using Meso Scale Discovery (Rockville) electrochemiluminescence immunoassays (S‐PLEX Proinflammatory Panel 1: interleukin 6 [IL‐6], IL‐10, IL‐12p70, IL‐4, tumor necrosis factor α [TNF‐α], IL‐2, IL‐1β, interferon γ [IFN‐γ], IL‐17A). Assays were run according to manufacturer's instructions; all samples were processed in duplicate and excluded from the analysis if the mean coefficient of variation was greater than 25%.^44^

Variables derived from the FBC that index inflammation were calculated, including the neutrophil lymphocyte ratio (NLR—neutrophil count divided by lymphocyte count) and the systemic inflammatory index (SII—neutrophil count times platelet count divided by lymphocyte count).^45^

### Statistical Analysis

Shapiro–Wilk tests were used to assess normality of the data. For group comparisons of demographical and clinical variables, continuous variables were compared using *t* tests or Mann‐Whitney *U* test (for parametric and nonparametric data, respectively), and categorical variables were compared using χ^2^ tests. Comparisons of ^11^C‐PK11195 BP_ND_ and blood/CSF markers between constipated and nonconstipated participants used Bayesian Student's *t* tests. The parameter BF_10_ (known as the Bayes factor) that we report is a measure of the evidence in favor of one hypothesis (H1) over another (H0), a particularly useful approach when small samples are compared, as it reduces the danger of false positives and negatives common to purely frequentist analyses.^29^ Relationships between regional ^11^C‐PK11195 BP_ND_, clinical measures, and serum/CSF markers were assessed using partial correlations adjusted for age and sex, given that there is an age‐dependent increase in ^11^C‐PK11195 BP_ND_, and age and sex differences in immune markers and constipation prevalence.^4^, ^19^, ^46^, ^47^ Association between clinical measures (GIDS‐PD, MDS‐UPDRS‐III, and ACE‐III) were assessed using Kendall tau rank correlations. SPSS and Graphpad Prism were used for statistical analyses and figures, respectively. Results are presented as mean ± standard deviation (SD). Analyses were two tailed, with significance considered at *P* < 0.05 for frequentist approaches and BF_01_ < 0.33 for Bayesian analysis.^48^

#### Data Sharing

Data are available upon request to the authors by qualified researchers. Requests will be considered on a case‐by‐case basis, assessing the feasibility and appropriateness of the proposed study and the capacity to maintain the required levels of data security, consistent with the original approved research ethics documentation and the patient information sheet that was the basis of consent obtained.

## Results

### Sample Characteristics

A total of 27 participants with PD underwent clinical assessment, ^11^C‐PK11195 brain PET‐MR, and completed the GIDS‐PD. A summary of demographic and clinical characteristics of the participants stratified by constipation status is presented in Table 1. There were no statistical differences between groups in terms of sex, disease duration and motor severity, LEDD, and cognitive scores. Constipated PD patients were older and had higher depression scores.

**TABLE 1:** Baseline demographic and clinical characteristics of Parkinson's disease (PD) cases stratified by constipation status (GIDS‐PD constipation ≥ 9)

	PD constipated (n = 11)	PD nonconstipated (n = 16)	*P*‐value
Sex (% male)	63.6%	68.8%	0.782
Age (y)	71.8 ± 6.3 (56–77)	64.7 ± 6.3 (51–77)	0.005*
Time from PD diagnosis (years)	1.9 ± 0.9 (0–3)	1.3 ± 0.8 (0–2)	0.089
H&Y stage (% of H&Y = 2)	72.7% (1–3)	81.3% (1–3)	0.617
MDS‐UPDRS‐III	30.4 ± 11.5 (8–46)	26.3 ± 6.9 (12–39)	0.368
LEDD (mg)	420.5 ± 172.1 (275–800)	338.4 ± 108.6 (100–550)	0.342
ACE‐III	91.8 ± 6.5 (80–99)	95.8 ± 3.7 (89–100)	0.080
GIDS‐PD constipation	17.1 ± 6.0 (9–31)	3.1 ± 1.9 (1–8)	<0.001*
GIDS‐PD bowel irritability	2.8 ± 3.1 (0–9)	1.4 ± 1.6 (0–6)	0.272
GIDS‐PD upper GI	3.2 ± 2.8 (0–8)	2.4 ± 2.3 (0–7)	0.544
GDS‐15	4.2 ± 2.2 (1–9)	1.6 ± 1.7 (0–5)	0.004*
CIRS	4.1 ± 1.9 (2–8)	2.9 ± 1.6 (1–6)	0.110
Cardiovascular disease	27.3%	25.0%	0.895

*Note*: Values shown are mean ± standard deviation and range. *, **, and **** denote *P* ≤ 0.05, *P* ≤ 0.01, and *P* ≤ 0.0001, respectively.

Abbreviations: H&Y, Hoehn and Yahr; MDS‐UPDRS‐III, Movement Disorders Society–Sponsored Unified Parkinson's Disease Rating Scale Part III; LEDD, levodopa‐equivalent daily dose; ACE‐III, Addenbrooke's Cognitive Examination III; GIDS‐PD, Gastrointestinal Dysfunction Scale for Parkinson's disease; GI, gastrointestinal; GDS‐15, Geriatric Depression Scale (15 item); CIRS, Cumulative Illness Rating Scale (number of organ systems affected).

### Relationship Between Constipation and ^11^C‐PK11195 BP_ND_

**FIG. 1:**
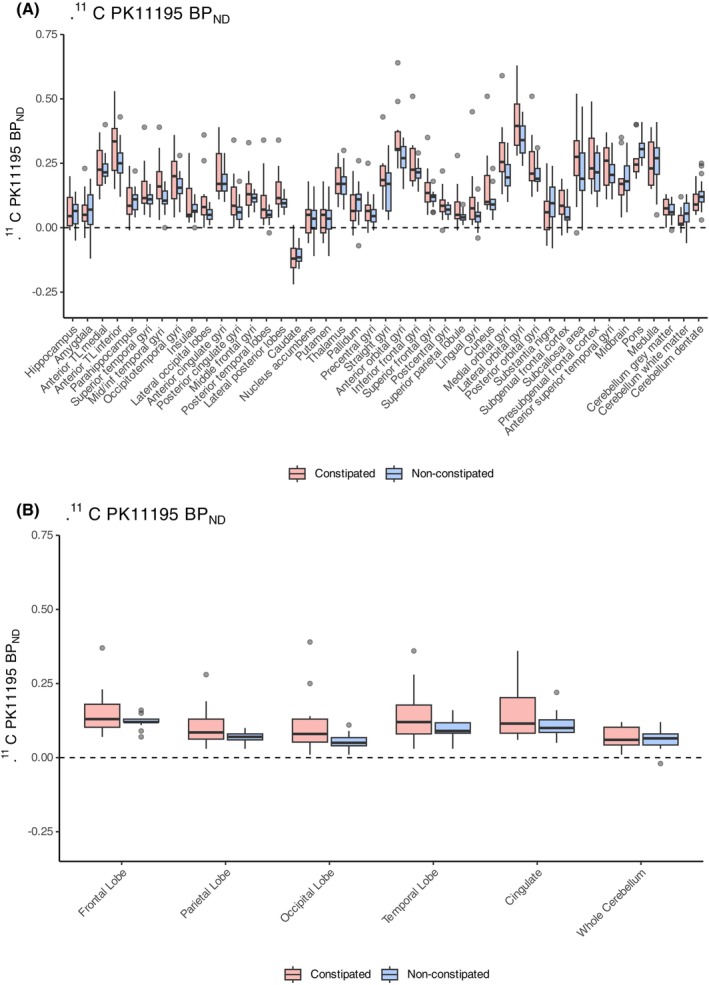
^11^C‐PK11195 BP_ND_ in constipated (n = 11) versus nonconstipated Parkinson's disease (PD) (n = 16) participants in regions of interest (**A**) and lobes (**B**). The box plots show the median and the interquartile range, with whiskers and outliers according to the Tukey approach. BP_ND_, nondisplaceable binding potential; TL, temporal lobe. [Color figure can be viewed at wileyonlinelibrary.com]

When participants were stratified by constipation status (GIDS‐PD constipation ≥9 vs. <9), although there were no significant between‐group differences in regional or whole‐brain ^11^C‐PK11195 BP_ND_, there was an overall trend toward constipated PD participants having higher PK11195 BP_ND_ across multiple regions (Fig. 1).

**FIG. 2:**
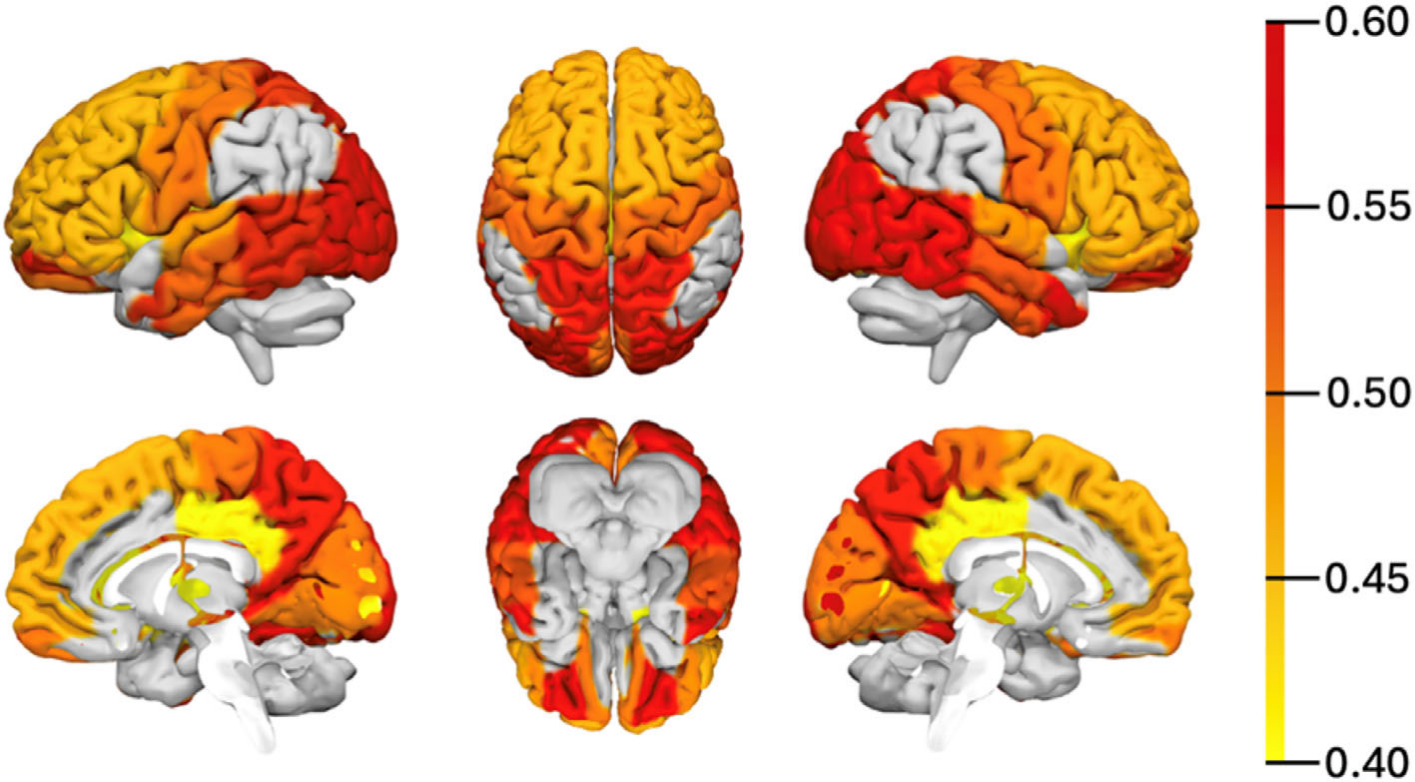
Regions of interest (ROIs) with significant partial correlations (*P* < 0.05) between regional ^11^C‐PK11195 BP_ND_ and GIDS‐PD constipation scores, adjusted for age and sex (n = 27). Color bar denotes the strength of the partial correlation, with red being the highest correlation strength. BP_ND_, nondisplaceable binding potential; GIDS‐PD, Gastrointestinal Dysfunction Scale for Parkinson's disease. [Color figure can be viewed at wileyonlinelibrary.com]

GIDS‐PD constipation scores were positively correlated with whole‐brain gray matter ^11^C‐PK11195 BP_ND_ (*r*[23] = 0.53, *P* = 0.007, adjusted for age and sex), and higher GIDS‐PD constipation scores were associated with higher ^11^C‐PK11195 BP_ND_ in the frontal, parietal, temporal, and occipital lobes (*r*[23] = 0.52, *P* = 0.007; *r*[23] = 0.58, *P* = 0.003; *r*[23] = 0.60, *P* = 0.002; *r*[23] = 0.52, *P* = 0.007) and multiple subcortical and cortical regions, with the strongest correlations found in the anterior orbital gyri (*r*[23] = 0.59, *P* = 0.002) and in the lateral occipital lobe (*r*[23] = 0.59, *P* = 0.002) (Fig. 2; Table S1).

Within the GIDS‐PD constipation subscore, comprising item 1 (low bowel frequency), item 2 (straining), item 3 (hard stools), and item 4 (incomplete evacuation), all but item 3 had multiple significant associations with ^11^C‐PK11195 BP_ND_, particularly GIDS‐PD item 4, which showed the strongest correlations with ^11^C‐PK11195 BP_ND_ in whole‐brain gray matter (*r*[24] = 0.64, *P* = 0.001) and the lateral occipital lobe (*r*[23] = 0.70, *P* < 0.001), among other cortical and noncortical ROIs (Supplementary Materials—File S1). There were no significant associations between regional ^11^C‐PK11195 BP_ND_ and other GIDS‐PD subscores (GIDS‐PD bowel irritability and GIDS‐PD upper GI).

To investigate whether associations between GIDS‐PD constipation and neuroinflammation were confounded by disease severity or depression, we performed partial correlations, adjusting for age, sex, motor severity (MDS‐UPDRS‐III), and depression (GDS‐15). These associations largely remained significant, with the strongest correlation between GIDS‐PD constipation scores and ^11^C‐PK11195 BP_ND_ in the anterior orbital gyri (*r*[21] = 0.56, *P* = 0.006) (Table S2).

### Relationship between ^11^C‐PK11195 BP_ND_ and Clinical Measures of Motor and Cognitive Severity

Partial correlations adjusting for age and sex showed that lower cognitive scores were associated with higher ^11^C‐PK11195 BP_ND_ in the occipital lobe (*r*[23] = −0.43, *P* = 0.030), particularly the lateral occipital lobe (*r*[23] = −0.44, *P* = 0.029) and the cuneus (*r*[23] = −0.48, *P* = 0.016), but not in whole‐brain gray matter (Supplementary Materials—File S2). Higher motor severity (MDS‐UPDRS‐III) was associated with higher ^11^C‐PK11195 BP_ND_ in whole‐brain gray matter and all lobes (0.40 < *r*[23] < 0.58, 0.002 < *P* < 0.048) and multiple cortical and noncortical regions (Supplementary Materials—File S2).

Kendal tau correlations showed that GIDS‐PD constipation scores were not associated with cognitive performance (ACE‐III total score) or motor severity in this cohort (MDS‐UPDRS‐III) (*P* > 0.355).

### Relationship Between Constipation and Peripheral and CSF Immune Markers

A total of 24 participants (13 constipated and 11 nonconstipated) met inclusion criteria (3 were excluded due to a vaccination in the preceding 2 weeks) and donated a blood sample; 11 of these participants underwent a lumbar puncture for CSF collection (4 constipated and 7 nonconstipated).

**FIG. 3:**
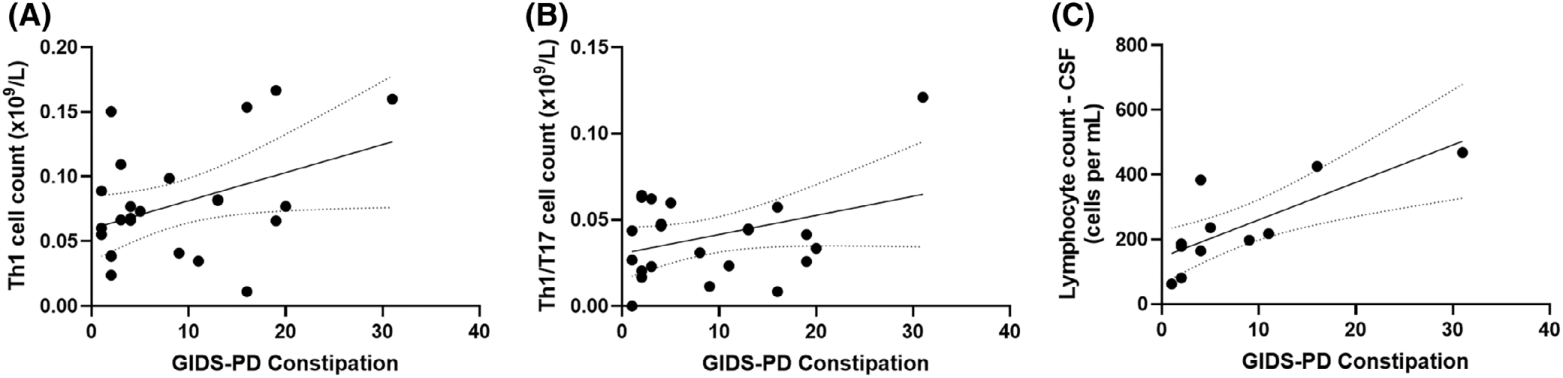
Correlations, adjusted for age and sex, between GIDS‐PD constipation scores and peripheral Th1 cell counts (*r*[20] = 0.47, *P* = 0.028) (**A**), peripheral Th17‐like Th1 cell counts (*r*[20] = 0.50, *P* = 0.017) (**B**), and higher cerebrospinal fluid (CSF) total lymphocyte counts (*r*[7] = 0.82, *P* = 0.007) (**C**).

Partial correlation analysis adjusted for age and sex showed that higher GIDS‐PD constipation scores were associated with higher Th1 cell counts (*r*[20] = 0.47, *P* = 0.028), Th17‐like Th1 cell counts (*r*[20] = 0.50, *P* = 0.017) in the periphery, and higher CSF total lymphocyte counts (*r*[7] = 0.82, *P* = 0.007) (Fig. 3). No significant correlations were found between GIDS‐PD constipation and SII, NLR (neutrophil‐to‐lymphocyte ratio), cytokines, and blood total lymphocyte counts.

## Discussion

Our study showed that constipation severity in early PD is associated with both changes in the peripheral immune cell profile and widespread brain inflammation, suggesting a possible inflammatory mechanism underlying the association between constipation and faster motor and cognitive progression in PD.

Previous studies using ^11^C‐PK11195 brain PET have reported increased ^11^C‐PK11195 binding, suggesting neuroinflammation in both early and later stages of PD.^28^, ^29^ The relationship between neuroinflammation and constipation has not been previously explored, but MRI‐based functional connectivity studies have shown changes in connectivity and in white and gray matter volume in non‐PD people with constipation compared to healthy controls, particularly in the anterior cingulate cortex, precentral gyrus, insula, and thalamus.^49^, ^50^, ^51^, ^52^, ^53^, ^54^ Our findings showed correlations between GIDS‐PD constipation scores and widespread neuroinflammation in early PD, and although it is not possible to differentiate the underlying etiologies of constipation (eg, delayed colonic motility, autonomic defecatory dysfunction, and microbial alternations), subitem analysis showed that incomplete evacuation scores had the strongest association with ^11^C‐PK11195 BP_ND_, suggesting that some features of constipation may be more strongly associated with inflammation than others. This is in keeping with previous non‐PD studies that reported that sensation of incomplete evacuation as a constipation feature correlates with cortical thickness in the supplementary motor area,^54^ higher low‐frequency fluctuation in the orbitofrontal cortex,^55^ and fractional anisotropy of the thalamus postcentral and thalamus–hippocampal gyrus tracts.^53^ Zheng et al, in a resting state functional MRI (fMRI) study of 106 PD patients with constipation and 73 PD patients without, reported higher amplitude of low‐frequency fluctuations in the right dorsal pons, cerebellum, and in the right insula in patients with constipation compared to those without.^56^ In our study, the strongest correlations between constipation scores and ^11^C‐PK11195 BP_ND_ were observed in the orbital gyri and in the occipital lobe, ROIs that have been implicated in cognitive impairment in PD.^29^, ^57^, ^58^, ^59^

Our study found a significant association between ^11^C‐PK11195 BP_ND_ and cognitive function, corroborating a previous study by our group, in which we found increased regional ^11^C‐PK11195 BP_ND_ in early PD cases at high versus low dementia risk, and a significant correlation between whole‐brain gray matter ^11^C‐PK11195 BP_ND_ and ACE‐III scores.^29^ In our study, GIDS‐PD constipation scores were not associated with ACE‐III. It is possible that our analysis was underpowered (by a limited sample size and ACE‐III score range) to detect a cross‐sectional relationship between constipation scores and cognition in the whole group. However, studies with bigger sample sizes have also failed to show cross‐sectional association between constipation and cognitive scores, while showing an association between constipation and faster progression to cognitive impairment and PD dementia.^5^, ^36^ It is possible that the detrimental impact of constipation may be incremental and more relevant to future development of cognitive impairment and dementia rather than current cognitive state.

^11^C‐PK11195 BP_ND_ also correlated with higher MDS‐UPDRS motor scores in our study, consistent with the findings of Ouchi et al (2005).^46^ It is possible that the correlations found between constipation and ^11^C‐PK11195 BP_ND_ are, to some extent, attributable to worse disease severity, so we adjusted for MDS‐UPDRS scores in our analysis, and associations between GIDS‐PD constipation scores and ^11^C‐PK11195 BP_ND_ remained significant. Furthermore, the constipated and nonconstipated groups in our study did not differ in PD duration or severity.

Our study also showed that constipation severity was associated with peripheral immune changes and with CSF lymphocyte count, supporting the hypothesis that peripheral immune activation and transition of peripheral immune cells into the CNS may mediate the relationship between constipation and neuroinflammation. A previous study has demonstrated that activated T lymphocytes in the CSF were increased in patients with PD compared to controls,^60^ and postmortem studies in PD have shown infiltration of CD4+ T lymphocytes into the brain parenchyma of people with PD and PDD compared to controls.^24^ Our study is the first to observe an elevated lymphocyte count in the CSF associated with constipation in PD. Some studies have reported that lower blood lymphocyte count is associated with the risk of developing PD,^61^ clinical presentation,^62^ and gut dysfunction.^63^ Neuropathological and in vivo studies have shown lymphocyte infiltration and increased blood–brain barrier permeability in PD, and it has been proposed that a reduction in blood lymphocytes in PD is consequential to their migration to the CNS.^62^

Peripheral T‐cell changes in healthy aging and PD are well documented, but less is known about their association to clinical features. Some studies have shown associations between PD motor severity, cognitive scores, or disease duration and CD3, CD4, and CD8 counts,^64^, ^65^, ^66^, ^67^ but these findings are not consistent.^47^, ^64^ Interestingly, people with functional constipation have increased CD3^+^ and CD4^+^ counts, which decline following constipation treatment, suggesting that their elevated count is secondary to constipation.^9^ In a study with 102 patients with PD, Chen et al^68^ showed that peripheral counts of Th17 and Treg cells in patients with PD and constipation (n = 79) were significantly higher than in those without constipation (n = 23). We observed a positive correlation between constipation and Th1 cell counts and Th17‐like Th1 cell counts. Given that Th1 and Th17 have been described to have a detrimental impact in neurodegeneration in PD,^69^ it is possible that the deleterious effect of constipation on PD progression is mediated via activation of these immune subsets in the gut. In a recent review, Campos‐Acuña et al^70^ proposed a hypothesis that T‐cell–driven inflammation, which is thought to contribute to dopaminergic neurodegeneration in PD, is triggered in the gut mucosa and highlighted the particular role of inflammatory effector CD4^+^ T‐cell subsets Th1 and Th17 in gut inflammation. The peripheral immune system is believed to contribute to PD progression through lymphocyte infiltration into the CNS in PD.^13^ There is evidence to suggest that constipation induces inflammatory processes,^9^ but it is equally possible that constipation may be secondary to local GI inflammation, which is associated with low‐grade systemic inflammation^10^, ^11^ and, in turn, CNS inflammation. The contribution of constipation in peripheral and CNS inflammatory processes in PD merits further investigation.

Several other limitations of this study should be considered when interpreting the results. Given that constipation is often underreported in PD,^71^ objective measures of constipation would provide a better understanding of its relationship with neuroinflammation, and future studies in our group will address this. The use of ^11^C‐PK11195 as a marker of neuroinflammation presents limitations due to its poor signal‐to‐noise ratio and low blood–brain barrier penetration.^59^ However, although second‐generation TSPO tracers with higher signal‐to‐noise ratio have been developed, unlike ^11^C‐PK11195, they are associated with genetically determined variability in binding affinity to TSPO, which adversely affects recruitment and statistical power. Moreover, recent evidence supports ^11^C‐PK11195 as a microglial biomarker, validating its use as a marker of neuroinflammation in PD.^59^ Additionally, there is now substantial evidence suggesting that the gut microbiota can modulate the host's immune system and regulate microglia throughout the lifespan.^72^ Unfortunately, fecal sampling was not available to determine whether certain microbiota species were independently associated with ^11^C‐PK11195 binding. Also, although we accounted for comorbidity scores and cardiovascular disease, we did not have data on the degree of small‐vessel disease (SVD), which may have contributed independently to increased ^11^C‐PK11195 binding. Finally, data were collected from a single‐center, cross‐sectional study with a small sample size, which limits the generalizability of our findings. However, our study provides novel data contributing to our understanding of the gut–brain axis. Further studies in larger cohorts are currently underway to validate our findings.

## Author Roles

(1) Research Project: A. Conception, B. Organization, C. Execution; (2) Statistical Analysis: A. Design, B. Execution, C. Review and Critique; (3) Manuscript Preparation: A. Writing of the First Draft, B. Review and Critique.

M.C.: 1A, 1B, 2A, 2B, 3A.

J.C.G.: 1B, 1C, 2C, 3B.

A.R.D.P.: 1C, 2C, 3B.

L.R.B.S.: 1C, 2C, 3B.

T.D.F.: 1C, 2C, 3B.

Y.T.H.: 1C, 3B.

J.H.: 1C, 3B.

L.K.: 1C, 3B.

C.H.W.G.: 1A, 1B, 1C, 2C, 3B.

## Financial Disclosures of All Authors (for the Past 12 Months)

The authors declare that they have no known competing financial interests or personal relationships that could have influenced the work reported in this paper. MC has received grant support from the Evelyn Trust and the NIHR Cambridge Biomedical Research Centre (BRC‐1215‐20014). JCG has received financial support from the Cambridge Centre for Parkinson‐Plus. CHWG holds a RCUK/UKRI Research Innovation Fellowship awarded by the Medical Research Council; has received grant support from the Cambridge Centre for Parkinson‐Plus, the NIHR Cambridge Biomedical Research Centre (BRC‐1215‐20014), the Michael J. Fox Foundation, the Evelyn Trust, Cure Parkinson's and Parkinson's UK; and has received consultancy payments from Evidera, Inc./GlaxoSmithKline.

## Supporting information


**Table S1.** Significant partial correlations between GIDS‐PD constipation scores and regional ^11^C‐PK11195 BP_ND_ adjusted for age and sex. BP_ND_, nondisplaceable binding potential; GIDS‐PD, Gastrointestinal Dysfunction Scale for Parkinson's disease.

**Table S2.** Significant partial correlations between GIDS‐PD constipation subscore and regional ^11^C‐PK11195 BP_ND_ adjusted for age, sex, MDS‐UPDRS and GDS‐15 scores. BP_ND_, nondisplaceable binding potential; GDS‐15, Geriatric Depression Scale (GDS‐15). GIDS‐PD, Gastrointestinal Dysfunction Scale for Parkinson's disease; MDS‐UPDRS‐III, Movement Disorder Society–Sponsored Unified Parkinson's Disease Rating Scale Part III.

**Data S1.** Supporting information 1.

**Data S2.** Supporting information 2.

## Data Availability

The data that support the findings of this study are available from the corresponding author upon reasonable request.

## References

[1] Adams‐Carr K , Bestwick J , Shribman S , Lees A , Shrag A , Noice A . Constipation preceding Parkinson's disease: a systematic review and meta‐analysis. J Neurol Neurosurg Psychiatry 2016;87:710–716. 10.1136/jnnp-2015-311680 26345189

[2] Camacho M , Macleod AD , Maple‐Grødem J , et al. Early constipation predicts faster dementia onset in Parkinson's disease. NPJ Parkinsons Dis 2021;7(1):45. 10.1038/s41531-021-00191-w 34039994 PMC8154963

[3] Jones JD , Rahmani E , Garcia E , Jacobs JP . Gastrointestinal symptoms are predictive of trajectories of cognitive functioning in de novo Parkinson's disease. Parkinsonism Relat Disord 2020;72:7–12. 10.1016/j.parkreldis.2020.01.009 32058266 PMC7179075

[4] Picillo M , Palladino R , Erro R , et al. The PRIAMO study: age‐ and sex‐related relationship between prodromal constipation and disease phenotype in early Parkinson's disease. J Neurol 2020;268:0123456789. 10.1007/s00415-020-10156-3 PMC7880965 32809151

[5] Leta V , Urso D , Batzu L , et al. Constipation is associated with development of cognitive impairment in de novo Parkinson's disease: A longitudinal analysis of two international cohorts. J Parkinsons Dis 2021;11(3):1209–1219. 10.3233/JPD-212570 33843697

[6] Houser MC , Tansey MG . The gut‐brain axis: is intestinal inflammation a silent driver of Parkinson's disease pathogenesis? NPJ Parkinsons Dis 2017;3(3):1–9. 10.1038/s41531-016-0002-0 28649603 PMC5445611

[7] Forsyth CB , Shannon KM , Kordower JH , et al. Increased intestinal permeability correlates with sigmoid mucosa alpha‐synuclein staining and endotoxin exposure markers in early Parkinson's disease. PLoS One 2011;6(12):e28032. 10.1371/journal.pone.0028032 22145021 PMC3228722

[8] Bu J , Liu J , Liu K , Wang Z . Diagnostic utility of gut α‐synuclein in Parkinson's disease: A systematic review and meta‐analysis. Behav Brain Res 2019;364:340–347. 10.1016/j.bbr.2019.02.039 30802532

[9] Khalif IL , Quigley EMM , Konovitch EA , Maximova ID . Alterations in the colonic flora and intestinal permeability and evidence of immune activation in chronic constipation. Dig Liver Dis 2005;37(11):838–849. 10.1016/j.dld.2005.06.008 16169298

[10] Ohama T , Hori M , Ozaki H . Mechanism of abnormal intestinal motility in inflammatory bowel disease: how smooth muscle contraction is reduced? J Smooth Muscle Res 2007;43(2):43–54. 10.1540/JSMR.43.43 17598957

[11] de Jong PR , González‐Navajas JM , Jansen NJG . The digestive tract as the origin of systemic inflammation. Critical Care 2016;20(1):1–12. 10.1186/S13054-016-1458-3 27751165 PMC5067918

[12] Reale M , Iarlori C , Thomas A , Gambi D , Perfetti B , di Nicola M , Onofrj M . Peripheral cytokines profile in Parkinson's disease. Brain Behav Immun 2009;23(1):55–63. 10.1016/j.bbi.2008.07.003 18678243

[13] Williams‐gray CH , Wijeyekoon R , Yarnall AJ , et al. Serum immune markers and disease progression in an incident Parkinson's disease cohort (ICICLE‐PD). Mov Disord 2016;31(7):995–1003. 10.1002/mds.26563 26999434 PMC4957620

[14] Grozdanov V , Bliederhaeuser C , Ruf WP , et al. Inflammatory dysregulation of blood monocytes in Parkinson's disease patients. Acta Neuropathol 2014;128(5):651–663. 10.1007/s00401-014-1345-4 25284487 PMC4201759

[15] Wijeyekoon RS , Kronenberg‐Versteeg D , Scott KM , et al. Peripheral innate immune and bacterial signals relate to clinical heterogeneity in Parkinson's disease. Brain Behav Immun 2020;87:1–16. 10.1016/j.bbi.2020.01.018 32006615 PMC7613010

[16] Saunders JA , Estes H , Kosloski LM , et al. CD4+ regulatory and effector/memory T cell subsets profile motor dysfunction in Parkinson's disease. J Neuroimmune Pharmacol 2012;7(4):927–938. 10.1007/s11481-012-9402-z 23054369 PMC3515774

[17] Williams‐Gray CH , Wijeyekoon RS , Scott KM , Hayat S , Barker RA , Jones JL . Abnormalities of age‐related T cell senescence in Parkinson's disease. J Neuroinflammation 2018;15(1):1–8. 10.1186/s12974-018-1206-5 29807534 PMC5972443

[18] Scott KM , Chong YT , Park S , et al. B lymphocyte responses in Parkinson's disease and their possible significance in disease progression. Brain Commun 2023;5(2):fcad060. 10.1093/braincomms/fcad060 36993946 PMC10042276

[19] Greenland JC , Holbrook J , Kahanawita L , Camacho M , Fryer TD , Hong YT , Williams‐Gray CH . Peripheral‐central immune crosstalk in Parkinson's disease and its association with clinical severity. Brain Behav Immun 2025;128:558–570. 10.1016/J.BBI.2025.04.028 40280259

[20] Stolzenberg E , Berry D , Yang D , et al. A role for neuronal alpha‐synuclein in gastrointestinal immunity. J Innate Immun 2017;9:456–463. 10.1159/000477990 28651250 PMC5865636

[21] Al‐Bachari S , Naish JH , Parker GJM , Emsley HCA , Parkes LM . Blood–brain barrier leakage is increased in Parkinson's disease. Front Physiol 2020;11:1–12. 10.3389/fphys.2020.593026 33414722 PMC7784911

[22] Ding XB , Wang XX , Xia DH , et al. Impaired meningeal lymphatic drainage in patients with idiopathic Parkinson's disease. Nat Med 2021;27(3):411–418. 10.1038/s41591-020-01198-1 33462448

[23] Brochard V , Combadière B , Prigent A , et al. Infiltration of CD4+ lymphocytes into the brain contributes to neurodegeneration in a mouse model of Parkinson disease. J Clin Invest 2009;119(1):182–192. 10.1172/JCI36470 19104149 PMC2613467

[24] Kouli A , Camacho M , Allinson K , Williams‐Gray CH . Neuroinflammation and protein pathology in Parkinson's disease dementia. Acta Neuropathol Commun 2020;8(211):1–19. 10.1186/s40478-020-01083-5 33272323 PMC7713145

[25] Mogi M , Harada M , Kondo T , Riederer P , Inagaki H , Minami M , Nagatsu T . Interleukin‐1 beta, interleukin‐6, epidermal growth factor and transforming growth factor‐alpha are elevated in the brain from parkinsonian patients. Neurosci Lett 1994;180(2):147–150. 7700568 10.1016/0304-3940(94)90508-8

[26] Loeffler DA , Camp DM , Conant SB . Complement activation in the Parkinson's disease substantia nigra: an immunocytochemical study. J Neuroinflammation 2006;3:1–8. 10.1186/1742-2094-3-29 17052351 PMC1626447

[27] Edison P , Ahmed I , Fan Z , et al. Microglia, amyloid, and glucose metabolism in parkinson's disease with and without dementia. Neuropsychopharmacology 2013;38(6):938–949. 10.1038/npp.2012.255 23303049 PMC3629382

[28] Gerhard A , Pavese N , Hotton G , et al. In vivo imaging of microglial activation with [11C](R)‐PK11195 PET in idiopathic Parkinson's disease. Neurobiol Dis 2006;21(2):404–412. 10.1016/j.nbd.2005.08.002 16182554

[29] Kouli A , Spindler LRB , Fryer TD , et al. Neuroinflammation is linked to dementia risk in Parkinson's disease. Brain 2024;147(3):923–935. 10.1093/brain/awad322 37757857 PMC10907093

[30] Schuitemaker A , Kropholler MA , Boellaard R , et al. Microglial activation in Alzheimer's disease: an (R)‐[11C]PK11195 positron emission tomography study. Neurobiol Aging 2013;34(1):128–136. 10.1016/J.NEUROBIOLAGING.2012.04.021 22840559

[31] Malpetti M , Rittman T , Jones PS , et al. In vivo PET imaging of neuroinflammation in familial frontotemporal dementia. J Neurol Neurosurg Psychiatry 2021;92(3):319–322. 10.1136/jnnp-2020-323698 33122395 PMC7892378

[32] Tondo G , Boccalini C , Caminiti SP , et al. Brain metabolism and microglia activation in mild cognitive impairment: A combined [18F]FDG and [11C]‐(R)‐PK11195 PET study. J Alzheimer's Dis 2021;80(1):433–445. 10.3233/JAD-201351 33579848

[33] Stær K , Iranzo A , Stokholm MG , et al. Cortical cholinergic dysfunction correlates with microglial activation in the substantia innominata in REM sleep behavior disorder. Parkinsonism Relat Disord 2020;81:89–93. 10.1016/j.parkreldis.2020.10.014 33099132

[34] Stokholm MG , Iranzo A , Østergaard K , et al. Extrastriatal monoaminergic dysfunction and enhanced microglial activation in idiopathic rapid eye movement sleep behaviour disorder. Neurobiol Dis 2018;115:9–16. 10.1016/J.NBD.2018.02.017 29522818

[35] Miyamoto T , Nakajima I , Arikawa T , Miyamoto M . Bowel movement frequency and difficult defecation using constipation assessment scale in patients with isolated REM sleep behavior disorder. Clin Park Relat Disord 2024;11:100269. 10.1016/J.PRDOA.2024.100269 39286572 PMC11404085

[36] Kong WL , Huang Y , Qian E , Morris MJ . Constipation and sleep behaviour disorder associate with processing speed and attention in males with Parkinson's disease over five years follow‐up. Sci Rep 2020;10(1):1–10. 10.1038/s41598-020-75800-4 33149217 PMC7643116

[37] Santos García D , García Roca L , de Deus Fonticoba T , et al. Constipation predicts cognitive decline in Parkinson's disease: results from the COPPADIS cohort at 2‐year follow‐up and comparison with a control group. J Parkinsons Dis 2022;12:315–331. 10.3233/JPD-212868 34602501

[38] Greenland JC , Cutting E , Kadyan S , Bond S , Chhabra A , Williams‐Gray CH . Azathioprine immunosuppression and disease modification in Parkinson's disease (AZA‐PD): a randomised double‐blind placebo‐controlled phase II trial protocol. BMJ Open 2020;10(11):e040527. 10.1136/bmjopen-2020-040527 PMC7684836 33234645

[39] Camacho M , Greenland JC , Williams‐Gray CH . The gastrointestinal dysfunction scale for Parkinson's disease. Mov Disord 2021;36(10):2358–2366. 10.1002/mds.28675 34133059

[40] Hudson HM , Larkin RS . Accelerated image reconstruction using ordered subsets of projection data. IEEE Trans Med Imaging 1994;13(4):601–609. 18218538 10.1109/42.363108

[41] Burgos N , Cardoso MJ , Thielemans K , et al. Attenuation correction synthesis for hybrid PET‐MR scanners: application to brain studies. IEEE Trans Med Imaging 2014;33(12):2332–2341. 10.1109/TMI.2014.2340135 25055381

[42] Manavaki R , Hong YT , Fryer TD . Brain MRI Coil Attenuation Map Processing for the GE SIGNA PET/MR: Impact on PET Image Quantification and Uniformity. EEE Nuclear Science Symposium and Medical Imaging Conference (NSS/MIC), Manchester, UK: IEEE (Institute of Electrical and Electronics Engineers); 2019:1–2. https://ieeexplore.ieee.org/document/9059867

[43] Lammertsma AA , Hume SP . Simplified reference tissue model for PET receptor studies. Neuroimage 1996;4:154–158. 10.1006/nimg.1996.0066 9345505

[44] Food and Drug Administration . Bioanalytical Method Validation Guidance for Industry Biopharmaceutics; 2018. http://www.fda.gov/Drugs/GuidanceComplianceRegulatoryInformation/Guidances/default.htmand/orhttp://www.fda.gov/AnimalVeterinary/GuidanceComplianceEnforcement/GuidanceforIndustry/default.htm.

[45] Hosseini S , Shafiabadi N , Khanzadeh M , et al. Neutrophil to lymphocyte ratio in parkinson's disease: a systematic review and meta‐analysis. BMC Neurol 2023;23(1):333. 10.1186/s12883-023-03380-7 37735638 PMC10512499

[46] Ouchi Y , Yoshikawa E , Sekine Y , Futatsubashi M , Kanno T , Ogusu T , Torizuka T . Microglial activation and dopamine terminal loss in early Parkinson's disease. Ann Neurol 2005;57(2):168–175. 10.1002/ANA.20338 15668962

[47] Niwa F , Kuriyama N , Nakagawa M , Imanishi J . Effects of peripheral lymphocyte subpopulations and the clinical correlation with Parkinson's disease. Geriatr Gerontol Int 2012;12(1):102–107. 10.1111/J.1447-0594.2011.00740.X 21929737

[48] Jeon M , De Boeck P . Decision qualities of Bayes factor and p value‐based hypothesis testing. Psychol Methods 2017;22(2):340–360. 10.1037/met0000140 28594227

[49] Peihong M , Tao Y , Zhaoxuan H , et al. Alterations of White Matter Network Properties in Patients With Functional Constipation. Front Neurol 2021;12:627130. 10.3389/FNEUR.2021.627130/BIBTEX 33841301 PMC8024587

[50] Yin T , He Z , Ma P , et al. Aberrant functional brain network dynamics in patients with functional constipation. Hum Brain Mapp 2021;42(18):5985–5999. 10.1002/HBM.25663 34533251 PMC8596972

[51] Jia Z , Li G , Hu Y , et al. Brain structural changes in regions within the salience network in patients with functional constipation. Brain Imaging Behav 2022;16(4):1741–1748. 10.1007/S11682-022-00648-3/TABLES/2 35278159

[52] Liu L , Hu C , Hu Y , et al. Abnormalities in the thalamo‐cortical network in patients with functional constipation. Brain Imaging Behav 2021;15:630–642. 10.1007/s11682-020-00273-y 32314199

[53] Zhang Z , Hu Y , Lv G , et al. Functional constipation is associated with alterations in thalamo‐limbic/parietal structural connectivity. Neurogastroenterol Motil 2021;33(12):e13992. 10.1111/NMO.13992 33073892

[54] Hu C , Liu L , Liu L , et al. Cortical morphometry alterations in brain regions involved in emotional, motor‐control and self‐referential processing in patients with functional constipation. Brain Imaging Behav 2020;14(5):1899–1907. 10.1007/S11682-019-00133-4 31218532

[55] Zhu Q , Cai W , Zheng J , et al. Distinct resting‐state brain activity in patients with functional constipation. Neurosci Lett 2016;632:141–146. 10.1016/J.NEULET.2016.08.042 27569716

[56] Zheng JH , Sun WH , Ma JJ , et al. Resting‐state functional magnetic resonance imaging in patients with Parkinson's disease with and without constipation: a prospective study. Clin Auton Res 2022;32(1):51–58. 10.1007/S10286-022-00851-8/FIGURES/2 35059875

[57] Abe Y , Kachi T , Kato T , et al. Occipital hypoperfusion in Parkinson's disease without dementia: correlation to impaired cortical visual processing. J Neurol Neurosurg Psychiatry 2003;74(4):419–422. 10.1136/JNNP.74.4.419 12640053 PMC1738406

[58] Kuhl DE , Minoshima S , Fessler JA , et al. In vivo mapping of cholinergic terminals in normal aging, Alzheimer's disease, and Parkinson's disease. Ann Neurol 1996;40(3):399–410. 10.1002/ANA.410400309 8797529

[59] Zhang PF , Gao F . Neuroinflammation in Parkinson's disease: a meta‐analysis of PET imaging studies. J Neurol 2022;269(5):2304–2314. 10.1007/S00415-021-10877-Z 34724571

[60] Schröder JB , Pawlowski M , Meyer zu Hörste G , et al. Immune cell activation in the cerebrospinal fluid of patients with Parkinson's disease. Front Neurol 2018;9:1081. 10.3389/fneur.2018.01081 30619041 PMC6305582

[61] Jensen MP , Jacobs BM , Dobson R , et al. Lower lymphocyte count is associated with increased risk of Parkinson's disease. Ann Neurol 2021;89(4):803–812. 10.1002/ana.26034 33527442 PMC9012149

[62] Grillo P , Sancesario GM , Bovenzi R , et al. Neutrophil‐to‐lymphocyte ratio and lymphocyte count reflect alterations in central neurodegeneration‐associated proteins and clinical severity in Parkinson disease patients. Parkinsonism Relat Disord 2023;112:105480. 10.1016/J.PARKRELDIS.2023.105480 37290213

[63] Bissacco J , Bovenzi R , Conti M , et al. Gastrointestinal dysfunction bears on the clinical‐biological profile of Parkinson's disease. Mov Disord Clin Pract 2024;12:497–503. 10.1002/MDC3.14319 39704323 PMC11998696

[64] Xiao Y , Wei Q , Ou R , et al. Association between peripheral adaptive immune markers and disease progression in Parkinson's disease. J Neurol 2023;270(9):4444–4450. 10.1007/s00415-023-11790-3 37278914 PMC10243250

[65] Bhatia D , Grozdanov V , Ruf WP , Kassubek J , Ludolph AC , Weishaupt JH , Danzer KM . T‐cell dysregulation is associated with disease severity in Parkinson's disease. J Neuroinflammation 2021;18(1):250. 10.1186/s12974-021-02296-8 34717679 PMC8556877

[66] Chen Y , Qi B , Xu W , et al. Clinical correlation of peripheral CD4+‐cell sub‐sets, their imbalance and Parkinson's disease. Mol Med Rep 2015;12(4):6105–6111. 10.3892/mmr.2015.4136 26239429

[67] Magistrelli L , Storelli E , Rasini E , Contaldi E , Comi C , Cosentino M , Marino F . Relationship between circulating CD4+ T lymphocytes and cognitive impairment in patients with Parkinson's disease. Brain Behav Immun 2020;89:668–674. 10.1016/j.bbi.2020.07.005 32688028

[68] Chen Y , Yu M , Liu X , et al. Clinical characteristics and peripheral T cell subsets in Parkinson's disease patients with constipation. Int J Clin Exp Pathol 2015;8(3):2495. 26045755 PMC4440064

[69] González H , Contreras F , Pacheco R . Regulation of the neurodegenerative process associated to Parkinson's disease by CD4+ T‐cells. J Neuroimmune Pharmacol 2015;10(4):561–575. 10.1007/s11481-015-9618-9 26018603

[70] Campos‐Acuña J , Elgueta D , Pacheco R . T‐cell‐driven inflammation as a mediator of the gut‐brain Axis involved in Parkinson's disease. Front Immunol 2019;10(FEB):239. 10.3389/FIMMU.2019.00239 30828335 PMC6384270

[71] Knudsen K , Krogh K , Østergaard K , Borghammer P . Constipation in parkinson's disease: subjective symptoms, objective markers, and new perspectives. Mov Disord 2017;32(1):94–105. 10.1002/mds.26866 27873359

[72] Keane L , Clarke G , Cryan JF . A role for microglia in mediating the microbiota–gut–brain axis. Nat Rev Immunol 2025;2025:1–15. 10.1038/s41577-025-01188-9 40506470

