# Supplementary material for: Constipation Is Linked to Neuroinflammation in Early Parkinson's Disease
Source: Mov Disord. 2025 Nov 13;41(2):455–65. doi: 10.1002/mds.70102 (PMC12951264; doi:10.1002/mds.70102)
Supplement: Supplementary file 1 — Table S1. Significant partial correlations between GIDS‐PD constipation scores and regional 11C‐PK11195 BPND adjusted for age and sex. BPND, nondisplaceable binding potential; GIDS‐PD, Gastrointestinal Dysfunction Scale for Parkinson's disease. [file MDS-41-455-s002.docx]

**Supplementary Table 1.** Significant partial correlations between GIDS-PD Constipation scores and regional ^11^C-PK11195 BP_ND_ adjusted for age and sex. BP_ND_ – non-displaceable binding potential; GIDS-PD - Gastrointestinal Dysfunction Scale for Parkinson’s disease.

| **Region of interest** | **Correlation adjusted for age(df), *p*-value** | **Region of interest** | **Correlation adjusted for age(df), *p*-value** |
| --- | --- | --- | --- |
| Superior frontal gyri | r(23)=0.48, *p*=0.016 | Precentral gyri | r(23)=0.52, *p*=0.007 |
| Middle frontal gyri | r(23)=0.47, *p*=0.019 | Postcentral gyri | r(23)=0.53, *p*=0.007 |
| Inferior frontal gyri | r(23)=0.48, *p*=0.015 | Superior parietal lobule | r(23)=0.56, *p*=0.004 |
| Anterior orbital gyri | r(23)=0.59, *p*=0.002 | Lateral posterior parietal lobe | r(23)=0.57, *p*=0.003 |
| Medial orbital gyri | r(23)=0.51, *p*=0.009 | Superior temporal gyri | r(23)=0.50, *p*=0.011 |
| Lateral orbital gyri | r(23)=0.52, *p*=0.007 | Middle/inferior temporal gyri | r(23)=0.53, *p*=0.007 |
| Presubgenual frontal cortex | r(23)=0.47, *p*=0.017 | Posterior temporal lobe | r(23)=0.57, *p*=0.003 |
| Insula | r(23)=0.45, *p*=0.026 | Lateral occipital lobe | r(23)=0.59, *p*=0.002 |
| Putamen | r(23)=0.47, *p*=0.019 | Lingual gyri | r(23)=0.52, *p*=0.008 |
| Posterior cingulate gyri | r(23)=0.47, *p*=0.018 | Cuneus | r(23)=0.52, *p*=0.007 |
