# Supplementary material for: Constipation Is Linked to Neuroinflammation in Early Parkinson's Disease
Source: Mov Disord. 2025 Nov 13;41(2):455–65. doi: 10.1002/mds.70102 (PMC12951264; doi:10.1002/mds.70102)
Supplement: Supplementary file 2 — Table S2. Significant partial correlations between GIDS‐PD constipation subscore and regional 11C‐PK11195 BPND adjusted for age, sex, MDS‐UPDRS and GDS‐15 scores. BPND, nondisplaceable binding potential; GDS‐15, Geriatric Depression Scale (GDS‐15). GIDS‐PD, Gastrointestinal Dysfunction Scale for Parkinson's disease; MDS‐UPDRS‐III, Movement Disorder Society–Sponsored Unified Parkinson's Disease Rating Scale Part III. [file MDS-41-455-s001.docx]

**Supplementary Table 2.** Significant partial correlations between GIDS-PD Constipation subcore and regional ^11^C-PK11195 BPND adjusted for age, sex, MDS-UPDRS and GDS-15 scores. BP_ND_ – non-displaceable binding potential; GIDS-PD - Gastrointestinal Dysfunction Scale for Parkinson’s disease; MDS-UPDRS-III - MDS-Unified Parkinson’s Disease Rating Scale; Geriatric Depression Scale (GDS-15).

| **Region of interest** | **Correlations**  **adjusted for age, sex, MDS-UPDRS-III and GDS-15**  **(df), *p*-value** |
| --- | --- |
| Anterior orbital gyri | r(22)=0.55, *p*=0.006 |
| Medial orbital gyri | r(22)=0.43, *p*=0.038 |
| Lateral orbital gyri | r(22)=0.47, *p*=0.022 |
| Postcentral gyri | r(22)=0.42, *p*=0.040 |
| Superior parietal lobule | r(22)=0.47, *p*=0.020 |
| Lateral posterior parietal lobe | r(22)=0.48, *p*=0.018 |
| Superior temporal gyri | r(22)=0.41, *p*=0.045 |
| Middle/inferior temporal gyri | r(22)=0.46, *p*=0.024 |
| Posterior temporal lobe | r(22)=0.52, *p*=0.010 |
| Lateral occipital lobe | r(22)=0.51, *p*=0.011 |
| Lingual gyri | r(22)=0.42, *p*=0.043 |
| Cuneus | r(22)=0.42, *p*=0.042 |
| Frontal lobe | r(22)=0.43, *p*=0.037 |
| Parietal lobe | r(22)=0.49, *p*=0.015 |
| Occipital lobe | r(22)=0.51, *p*=0.010 |
| Temporal lobe | r(22)=0.44, *p*=0.030 |
| Whole brain grey matter | r(22)=0.45, *p*=0.028 |
